# Customary Legal Empowerment in Namibia and Ghana? Lessons about Access, Power and Participation in Non‐state Justice Systems

**DOI:** 10.1111/dech.12415

**Published:** 2018-05-04

**Authors:** Janine Ubink

## Abstract

Since the early 2000s, legal development cooperation has displayed an increasing willingness to engage with customary justice systems. However, this engagement is frequently problematic. External actors often lack knowledge about the different versions of customary law, the negotiable nature of customary justice and the power differentials involved in defining customary law. In customary justice systems, norms are defined and negotiated in administrative structures and dispute‐settlement institutions. Inclusion in these fora is therefore of paramount importance to improve the position of vulnerable groups. To illustrate the point, this article analyses two case studies of customary justice reform, respectively focusing on gender dimensions in northern Namibia and land management in Ghana. These case studies demonstrate that when programming ignores issues of power and empowerment, it will not have the hoped‐for positive impact on vulnerable groups.

## INTRODUCTION

Development practitioners involved in ‘rule of law’ building projects recently accepted the importance of customary justice systems (CJSs) for the majority of rural inhabitants in the global South. Their increasing willingness to engage with CJSs in rule of law building programmes is, however, often problematic. Donor and development agencies’ approaches and expectations do not easily tally with the complexity of CJSs. External actors often lack knowledge about the different versions of customary law, the negotiable nature of customary justice and the power differentials involved in defining customary law.

The unwritten, negotiable and relational nature of customary law is an important determinant for programming, as is the variety in normative beliefs and practices within customary communities. Customary norms are formulated, renegotiated and flexibly applied in administrative structures and dispute settlement institutions. Inclusion in these fora, where law is defined and negotiated, is therefore of paramount importance to improve the position of vulnerable groups. This requires paying attention to issues of representation and participation of marginalized community members in CJSs, and their ability to make use of these systems to uphold their rights and obtain outcomes that are fair and equitable.

To illustrate this point, this article analyses two case studies with very different approaches to reforming CJSs. In the first case study, Uukwambi Traditional Authority (TA) in northern Namibia tried to enhance the role of women in the CJS. Stimulated by the national government and by a clamour for change from society, the TA initiated changes in three arenas: several customary norms that were detrimental to women were altered, women's participation in traditional dispute settlement was encouraged and the number of female traditional leaders was increased. The measures adopted prompted positive change in customary practices. For example, the new female traditional leaders were well accepted by their communities and their administration assessed positively. In addition, the nature of traditional court meetings changed enormously, with more women present and actively participating. The majority of court users were satisfied with the performance of the court, and felt that men and women were treated equally and had an equal chance to obtain a fair decision or settlement; a new norm prohibiting relatives laying claim to the property of a widow on the death of her husband was widely known, and the practice gradually declined. These new measures led to significant changes in customary law with a substantially stronger role for Uukwambi women in the CJS.

In the second case study, a heavily donor‐sponsored Land Administration Programme (LAP) aimed to improve the quality of customary land administration in Ghana by introducing customary land secretariats. These were to enhance smallholders’ tenure security and customary actors’ accountability regarding land transactions. However, donors and government actors involved in LAP engaged with the customary land tenure system in a way that enhanced traditional leaders’ powers to administer and regulate communal land, without attention to the smallholders’ participation and representation or the need for the chiefs’ accountability. This allowed for elite capture of increasing land revenues — to the detriment of ordinary land users.

This article builds on six years of research into customary land management in Ghana (2002–08), including 15 months of fieldwork in peri‐urban Kumasi. A further two years of research into gender dimensions of customary law in northern Namibia (2012–14) included five months of fieldwork in Uukwambi Traditional Authority. Both studies dealt with CJSs and attempts to engineer change in these systems. Very different means were employed in these attempts at reform, with highly divergent outcomes regarding the people and groups that benefited and that lost out from the changes. The approach by Uukwambi TA enhanced the ability of a vulnerable group — women — to participate in and make use of the CJS. The result was stronger satisfaction with the system and improved protection of women's rights. The LAP excluded the vulnerable group that was intended to benefit from the programme — Ghana's smallholders — from interpreting, negotiating and administering customary norms and processes, which resulted in strong dissatisfaction with the CJS and its leaders, and a loss of smallholders’ rights to land and tenure security.

These two case studies thus aptly illustrate that programming that ignores relations of power and empowerment will not have the hoped‐for positive effect on vulnerable groups. Improving CJSs’ functioning and effectiveness requires a particular kind of legal empowerment — customary legal empowerment. This term emphasizes the importance of improving representation and participation of marginalized community members in CJSs, and their ability to make use of these systems to uphold their rights and obtain outcomes that are fair and equitable (Ubink and van Rooij, 2011: 17).

This article discusses debates in the field of legal development cooperation on effective programming and the merits of top‐down versus bottom‐up approaches, focusing on state justice mechanisms versus customary justice mechanisms. Thereafter, it presents the two illustrative case studies of gender and land customary law reform in Uukwambi and Ghana, and an analysis of the resulting (lack of) empowerment of marginalized groups. The article concludes that awareness of powers of representation and negotiation are of paramount importance when engaging with CJSs.

## RULE OF LAW IN A CONTEXT OF LEGAL PLURALISM

In the early 1990s, law regained an important role in the field of development cooperation. International donor organizations expanded programmes to enhance legislation and legal institutions in developing countries, so as to promote economic growth, good governance and human rights (Carothers, 2006; van Rooij, 2012; Trubek and Santos, 2006). Traditionally, these programmes emphasized state legislation and formal institutions, while development practitioners largely glossed over CJSs or considered them incompatible with the modern nation state, and a constraint on development (Chirayath et al., 2005). According to Sage and Woolcock (2006), this disregard stems from the belief that law must be uniform and monopolized by the state, and that development, whether economic or political, depends on this. The first wave of donor‐led legal reform projects in the 1990s thus focused on strengthening legislation and formal justice institutions, such as the judiciary, legislators, public prosecutors, police and prisons. Over the years, legal interventions in this ‘rule of law orthodoxy’ (Upham, 2002: 75) became increasingly criticized for their ineffectiveness in realizing development. Explanations included the top‐down character of law reform projects with minimal consideration of local contexts; donors’ state‐centred approach, with its focus on courts and law‐making processes, and tendency to ignore non‐state normative systems and resultant issues of legal pluralism; insufficient awareness of the inherent political nature of legal reform among development practitioners; and donors’ bureaucratic structures (van Rooij, 2012).

Responding to these criticisms, at the turn of the century, legal development cooperation shifted course towards a focus on the users of justice systems rather than legal institutions. Employing terms such as ‘access to justice’ and ‘legal empowerment’, donors now aimed to base programming on the poor's needs and preferences. This was to bring direct benefits to the poor, instead of a trickle‐down effect of enhanced justice systems (van Rooij, 2012: 287). While there are many definitions of these overlapping terms, essentially they refer to ensuring that legal and judicial outcomes are just and equitable and that justice systems (also) work for the poor and disadvantaged. This includes aspects of accessing the system and empowering vulnerable groups to use it to their advantage (Asian Development Bank, 2001; Golub, 2003; UNDP, 2005). ‘Bottom‐up approaches’ generally emphasize legal awareness, legal aid, alternative dispute resolution and stronger community organization (van Rooij, 2012: 293).

The increased focus on the poor also led to an increasing willingness to engage with CJSs, following from the realization that the lives of the poor majority in developing countries continue to be largely governed by customary norms and institutions, especially in the fields of marriage, family relations and access to natural resources (Harper, 2011b; Ubink and van Rooij, 2011). However, engaging with CJSs in rule of law programming is quite controversial. According to Sage and Woolcock (2006: 2–3), it is ‘fraught with its own concerns and unknowns’. Due to high levels of geographical variation and customary law's complex unwritten, negotiable and relational nature, it is difficult to gain adequate knowledge to design legal reform programmes. Harper (2011a) argues that the amount of research required to gain in‐depth knowledge of CJSs and local power relations makes for a labour‐intensive approach. It is not likely to sit well with expectations and approaches of many donors and development agencies. Sage and Woolcock (2006: 4–9) point out that development practitioners tend to see legal reform as a variant on other familiar technical problems — such as building roads and immunizing children — thereby overlooking that legal systems are social interventions that ‘draw their salience and strength from the acquiescence of those using them, becoming meaningful, actionable, and legitimate through idiosyncratic political and cultural processes’ (ibid.: 6). Moreover, the bureaucratic nature of development agencies, with their imperatives of measurable outcomes, quality control and working at scale, leave little leeway for differentiation on the basis of variances in local contexts and their inherent complexities. Furthermore, economists are dominant in large development organizations, contributing to a turn away from local specifics towards more generalizable models. This is problematic for legal reform generally, and particularly for engaging with CJSs, which is ‘messy, nuanced and context‐specific’ in nature (Harper et al., 2011: 173).

Central to CJSs’ complexity is the fact that multiple versions exist. Many countries have recorded customary law in textbooks, codifications or case law, and these recorded versions often differ substantially from living customary law (Oomen, 2005; Ubink, 2002–04). Even in living customary law, there often exist competing versions of norms within groups or communities, particularly in contexts of large social and economic transformations. This norm pluralism can be witnessed not only in normative statements, but also in variation in practices (Chanock, 1989; Ubink, 2008a). It is critical, therefore, which person or group is given, or is able to appropriate, the power to define the applicable customary norms. It is important to consider who is excluded and who is privileged. Limited understanding of the existing variations may lead to relying on representatives of one particular group — often male elders and traditional leaders. This may result in that group representing customary norms in their own interest, and downplaying versions that may benefit subaltern community members.

Even in cases where norms are not contested, the unwritten customary law is flexible, relational and negotiable in character. Customary norms are seldom used to determine directly who wins and who loses, but are rather used as a starting point for discussions towards mediated outcomes. This is connected to the more communal, interdependent character of small communities, and to the limited enforcement power of most CJSs — which emphasizes mediation and acceptance of the outcome by both parties and the wider community. Some see the negotiability and mediation as opening up access to justice, even for marginalized community members. Others, however, point out that in practice not everything is negotiable, and that some are in a better bargaining position than others (Peters, 2002; Ubink, 2008b; Woodhouse, 2003).

Interestingly, the two responses to the failures of the rule of law orthodoxy — bottom‐up approaches focusing on access to justice and legal empowerment, and increasing willingness to engage with CJSs — have not led to much awareness of access and power differentials within CJSs. It is almost as if development practitioners regard working with CJSs as automatically empowering and inherently bottom–up. Legal development actors, and the state and non‐state organizations they work with, often lack knowledge about the different versions of living customary norms, the negotiable nature of customary justice and the implications this has for engagement with CJSs. Their programming may alter power relations in the local community. Consciously, projects aim to empower marginalized groups, and doing so may decrease the relative local power base of original elites. However, fixating on traditional leaders and elders as community representatives and custodians of customary law easily leads to accepting elite representations of customary law. It also forges links between state institutions, and elite norms and institutions in the customary system. This process overlooks different, contested versions of law in the locality, and reinforces the subordinate position of marginalized groups. Power differentials may similarly be strengthened where the negotiable nature of customary law is not taken into account, and efforts subsequently fail to focus on harnessing weaker parties in the negotiated settlement processes (Ubink and van Rooij, 2011).

Elite power can also hinder legal reform activities when customary power holders are able to resist and co‐opt reforms. This is especially likely when the reforms are seen as a threat to the elite power base. The power dimension also informs the debate over local ownership of development activities. The quest for local legitimacy and acceptance leads development programming to work, as much as possible, through local actors. If purely internal processes are used, however, there is a risk of perpetuating power inequalities. As the power distribution plays a vital role in improving the functioning of customary justice, engaging with CJSs calls for a careful consideration of the local actors and their power differentials, and nuanced interventions to initiate or accentuate internal cultural discourse. This requires neither dealing solely with traditional leaders, nor alienating or ignoring them (Johnstone, 2011).

Based on this analysis of the role of power in CJSs and the peril of ignoring power relations in legal reform projects, it can be concluded that programming in customary justice needs to engage more with issues of power and empowerment (Harper, 2011b; Ubink, 2011a). Improving CJSs’ functioning and effectiveness requires a particular kind of legal empowerment — ‘customary legal empowerment’. This term emphasizes the importance of improving the representation and participation of marginalized community members in CJSs and their ability to make use of these systems to uphold their rights and obtain outcomes that are fair and equitable (Ubink and van Rooij, 2011: 17). This is corroborated by several studies. In Papua New Guinea, a project was found to be most successful in improving customary justice mechanisms for women where it focused on transferring dispute resolution skills to women, and women's inclusion as mediators (Johnstone, 2011). Similarly, in Rwanda, a legal reform project improved women's rights to land, through an increased role for women representatives in mediation (Lankhorst and Veldman, 2011). A project in Somaliland, which was largely conceptualized and driven by elders, fell short of its objectives because it did not include measures to enhance accountability and did not empower users to demand change (Simojoki, 2011). A comparative action research study of community land titling in Liberia, Mozambique and Uganda emphasizes that debating community by‐laws and constitutions in an unrestricted public forum opens up a space for women to challenge traditional rules that discriminate against them (Knight, 2011).

## ‘GENDER MAINSTREAMING’ CUSTOMARY JUSTICE IN NAMIBIA^1^

Structures and processes of customary justice are commonly portrayed as largely patriarchal and favouring the interests of men above those of women. This gender imbalance is visible in substantive customary norms, traditional leadership structures, and the operation of customary courts. The patriarchal principles of traditional justice operate to create a gender bias and ensure that major decisions on issues such as land allocations, inheritance and divorce are almost invariably taken by, and in favour of, men (Nyamu‐Musembi, 2003; Stewart, 2008; Whitehead and Tsikata, 2003).

Owambo, in the north of Namibia, is no exception. Research carried out in 1992–93 revealed that Owambo women had limited knowledge of, and lacked access to, the CJS. Many women perceived customary law and the customary judicial system as neglecting their concerns. They particularly complained that they were excluded from active participation in customary courts (Namibia Development Trust, 1993). In Owambo, as in many other areas of Namibia, the colonial period witnessed a profound change in gender relations. While women's role in pre‐colonial Owambo is contested,^2^ it is generally accepted that pre‐colonial societies afforded women more respect, protection and security than the colonial society did. For the purposes of colonial control and to ensure social order and stability, the colonial government aimed to strengthen the authority of male elders over women and young people, and thus entrenched women's disadvantages in traditional society. Tribal authorities, with the backing of, or even under instigation by, the colonial government, manipulated African customs to subordinate women (Becker, 1995; Hayes, 1998). Thus, specific features of customary rule were ‘revived out of their historical and social context’ (Ginwala, 1988: 50) to strengthen the already existing patriarchal oppression (SWAPO Women's Council, 1988).

The colonial rulers’ gender ideology also led to purging women traditional leaders and to excluding women from participation in traditional courts (Becker, 2001, 2005). Migrant (male) labour and the resulting monetization of the economy further concentrated power in the hands of men, as it weakened the financial position of women vis‐à‐vis men, and downgraded their domestic and agricultural work to ‘non‐paid’ activities. This resulted in women losing recognition as important producers (Becker, 1995: 97–98). Christianity and Western missionaries, with their model of the nuclear family and domesticity for women, deepened women's subordinate position in Owambo society (Becker, 2005). Although the Owambo gender ideologies are a rather recent innovation resulting from many deliberate — and unintentional — transformations during the colonial era, they are currently generally seen as ‘traditional’ (Ubink, 2013: 108).

To enhance the CJS's inclusiveness, the Uukwambi Traditional Authority — one of the seven TAs in Owambo — under leadership of its chief Herman Iipumbu, has initiated several changes over the last few decades. These address gender imbalances in all three interwoven domains of customary rule: leadership structures, court processes and substantive norms. The Uukwambi TA, and its chief in particular, has been actively promoting women's leadership since the 1980s, both in speeches and through appointing women at various levels of traditional leadership, including a woman deputy chief. While they are still heavily outnumbered by headmen, this has led to a significant increase in the percentage of women leaders.^3^ In 1993, at a Customary Law Workshop convened by the Owambo TAs, it was unanimously decided that women should be allowed to participate fully in traditional courts’ actions. In response to this, the Uukwambi TA communicated to all village heads that women were to be encouraged to participate actively in traditional court meetings and that each village needed to select a woman representative to act as the deputy to the village head. Another outcome of the Owambo Customary Law Workshop in 1993 was a recommendation that the councils of the various Owambo traditional communities change their customary norms to end ‘widow chasing’ or ‘property grabbing’. These terms refer to instances where widows and their children are being chased out of their houses, back to the widows’ matrilineal family, on the death of their husbands. At the workshop, it was decided that widows should be allowed to stay on their land, and without payment to the village head. In response to this recommendation, widows’ protection was included in the written laws of Uukwambi TA.

The real question obviously centres on the effect of all these well‐meant changes initiated by the Uukwambi TA and its chief. To what extent did these initiatives go beyond formal changes that de facto improved the rights and position of women? Did they genuinely empower women as leaders, as ‘judges’ in traditional courts, and as rights seekers? Fieldwork undertaken by the author in Owambo demonstrates that the performance of the new women traditional leaders is widely seen as satisfactory and largely equal to men's performance. The majority of traditional leaders and villagers still state that they believe men generally make better leaders than women, and that they prefer male leaders to female ones. While this displays the continued existence of gendered attitudes towards leadership, the data show that villagers, and particularly men, are more open to, and positive about, women leaders when they live in a village under female leadership. This is an important finding, as it indicates that men's opinions about gendered leadership — whether based on traditional values or preconceived opinions regarding character traits of men and women — undergo significant change as a result of exposure to successful female leadership (Ubink, 2013).

In stark contrast to what women reported in 1992/93, women's participation in traditional court meetings has become an accepted feature of Uukwambi cultural life at village, district and TA levels. In court observations, the author witnessed active participation of women as ‘judges’, parties, witnesses and members of the general audience. There was no discernible difference of style in behaviour and speech between men and women. This may not have been the case for all women, though, as several respondents stated that some, particularly elder women, still found it hard or inappropriate to speak up during court proceedings.

The vast majority of both female and male respondents reported that they felt they could actively participate in court proceedings and that men and women received equal treatment in the courts and had an equal chance to get a fair decision. There was a marked difference between respondents in villages with a headwoman and those with a headman. Female respondents were significantly more positive about the traditional court proceedings in female‐headed villages, in terms of overall performance; ability to participate in the proceedings; and the equal division of power among the sexes. Male respondents were slightly more positive about the overall performance of traditional courts in male‐headed villages, but indicated that the power division among the sexes was more equal and that they spoke up more easily in courts in female‐headed villages. Both male and female respondents were slightly more positive about equal treatment by courts where the traditional courts were headed by women. With regard to the changed norms on widows’ inheritance, qualitative interviews and the survey clearly demonstrated that the norms have become widely known and enforced in Uukwambi, and that cases of ‘property grabbing’ have reduced, both in traditional courts and at Communal Land Boards,^4^ and occur only seldom nowadays.^5^

## LAND ADMINISTRATION PROJECT GHANA^6^

Where the Uukwambi case study focuses on gender differences in customary systems, this land case rather displays elite co‐optation of customary law — traditional leaders profiting to the detriment of smallholders — and as such, highlights class differences in customary justice structures. In Ghana, land transactions have become increasingly monetized in recent years as a result of growing scarcity and increased land values. The new developments, and the changing values in land that they create, result in attempts to redefine land ownership and tenure, and contestation of rights to land. These processes have increasingly concentrated control of the economic benefits flowing from land in the hands of traditional chiefs, which has a direct negative effect on people's livelihoods and creates high tensions in many localities. It is against this background that the government of Ghana, in 2002, embarked on the Land Administration Project (LAP) Ghana — a long‐term programme with multi‐donor support, that aimed to provide greater certainty of land rights for ordinary users and greater efficiency and fairness in the land market (Ministry of Lands and Forestry, 2003; World Bank, 2003). One part of the project focused on enhancing the customary land sector's functioning.

The Ghanaian Constitution states that customary lands are to be managed by the appropriate traditional authority on behalf of, and in trust for, their people.^7^ It does not make more specific provisions on how customary lands should be managed by traditional authorities. In practice, increasing land values lead to widespread disputes over power to allocate rights in customary land and entitlements to the proceeds of these land allocations. Peri‐urban areas of Ghana, for instance, witness severe struggles between farmers and families on the one hand, and chiefs on the other, over the right to convert farmland into residential land. Chiefs allocate this land to outsiders through customary leases (Abudulai, 2002; Alden Wily and Hammond, 2001; Berry, 2002; Gough and Yankson, 2000; Kasanga and Kotey, 2001; Ubink, 2007). As a result of these allocations, the original land users, with weaker bargaining power, frequently lose their land, their employment and their income base. Traditional authorities display little accountability in using monies generated and most indigenous land users realize little or no benefit from leasing out land. They are rarely, and then inadequately, compensated for land loss and in most villages, only a meagre share of the revenue is used for community improvement. In other areas of Ghana, particularly in areas with high agricultural potential, comparable struggles over land and its proceeds can be witnessed between chiefs and community members (Amanor, 2005; Berry, 1997; Boni, 2006; Firmin‐Sellers, 1995; Fred‐Mensah, 2000; Hill, 1963; Lentz, 2006; Lund, 2006; Tonah, 2002). As such, the practice of customary land management in Ghana differs widely from the constitutional provision that puts the interest of the community first.

In 2003, the government of Ghana embarked upon the LAP, which targeted improvements in the state land sector and the customary land sector. The focus on enhancing customary land management was in line with the international trend in land policy in developing countries: to emphasize the importance of recognizing and building on customary tenure systems to achieve equitable land management and poverty reduction (Deininger and Binswanger, 1999; DFID, 1999; EU, 2004; World Bank, 2003). This policy trend is subject to critique because customary land rights are the outcomes of negotiations, struggles, disputes and implicit agreements embedded in social relations of family, kinship and community. These social relations are also inherently unequal, involving power relations between ordinary land users and customary authorities. The powers and opportunities of customary authorities to redefine customary ‘law’ in their own interests may increase as a result of formalizing customary tenure systems and institutions over which they exert significant influence and control (Anaafo, 2015; Bassett, 1993; Berry, 1993; Shipton and Goheen, 1992).

The LAP sought to enhance the functioning of customary land management through developing Customary Land Secretariats (CLSs) as efficacious land administration structures ‘with appropriate governance structures to assure institutionalised community‐level participation and accountability in the use of stool^8^ land and the revenue it generates’ (Ubink and Quan, 2008: 205). The CLSs’ objectives are to strengthen the customary authorities’ accountability in land management, in line with constitutional requirements. CLSs are to provide effective land management and to establish a unified, decentralized public record of land availability, use and transactions. The principal beneficiaries are expected to be the majority of people — for whom the current land administration system is effectively inoperable, due to the lack of transparency in the land allocation process, uncertain tenure rights, high costs, and slow, complex bureaucratic procedures (Toulmin et al., 2004).

Customary authorities in Ghana (and, for that matter, in many other countries) frequently do not manage lands in the interests of the holders of customary rights. This is because of the opportunities to generate revenues from sales and transactions in land. Therefore, LAP's twin goals — of greater certainty for ordinary land users, and gains in efficiency and fairness in the land market — could only be met if the CLS, as an institution, was designed to promote the land rights of smallholders. However, from the inception of LAP, it has been the government's clear political choice that CLSs should fall under the aegis of traditional authorities, rather than opting for more community‐based approaches to customary land management. By placing the CLSs under the aegis of the chiefs, LAP ignores that the notion of the ‘customary’ powers and rights of chiefs is loaded with political inventions and endorses the roles that chiefs were accorded in land administration in the colonial period — as if this were a timeless principle of customary tenure (Amanor, 2005: 110–11).

Guaranteeing security of title of small landowners in peri‐urban Ghana against powerful chiefs and elders requires clarifying the nature of usufructuary rights, and protecting these rights against the chiefs’ conversion drive (see Ministry of Lands and Forestry, 2003; Toulmin et al., 2004; World Bank, 2003). During the LAP conception and design process, however, there was no wide and open discussion of the chiefs’ role in stool land administration — including the chiefs’ tendency to behave like private landlords — or of the possible checks and balances the state could place on this administration (Alden Wily and Hammond, 2001).^9^ Quite the contrary, the overriding need to gain the support of chiefs in establishing CLSs has led to restraint in pushing for clarifying the rights of land users. At the inception of LAP, and prior to recruiting dedicated CLS development personnel, the government presented the idea of pilot CLSs to traditional leaders, as packages of equipment and technical support to help resource, and improve efficiency in, existing customary land management practices. As a result, in the words of one of the World Bank's specialists, ‘now they like the project because we do not prescribe anything’.^10^ According to Aryeetey et al. (2000), chiefs have been highly successful in getting their issues on the land reform agenda, in contrast to other groups such as tenants, migrant farmers, women and young people.

Through the CLS piloting process, LAP staff also had opportunities to draw up, discuss and introduce Memoranda of Understanding (MoUs) between the ministry and the chiefs, setting out the responsibilities on both sides. They could thus work towards establishing a wider regulatory framework for CLSs, which would be informed by the piloting process. However, the government has not made efforts to adapt model MoUs drafted by the CLS facilitation team, and have them signed as formal agreements between the ministry and the chiefs to govern the pilot‐CLS operations. LAP has even advised against using, in draft MoUs, language which might be interpreted by the chiefs as imposing requirements of accountability, disclosure of revenues or significant commitments of stool resources to supporting CLSs. The government has also not introduced a clear policy on the purpose and responsibilities attached to CLSs, and the parameters for establishing each pilot CLS remain somewhat ad hoc. What is clear is that, to secure the votes that the chiefs command, the government was unlikely to risk antagonizing the chiefs by requiring public disclosure of land revenues and accountability in their use, in line with the government's broader ‘policy of non‐interference’ in chieftaincy affairs (Ubink, 2008a: 96–98). According to DFID's rural livelihoods adviser, ‘Land reform is not the sort of thing you'd sensibly pursue, with the [upcoming] elections in your mind’.^11^ He also pointed out that LAP project staff have found it difficult to speak up when not backed by the minister.

This backdrop of governmental attitude and operation of the LAP Unit established under the Chief Director of the Ministry of Lands and Forestry, informs the operation of the CLSs. In their 2007 analysis of the functioning and impact of the first 10 pilot CLSs established since the end of 2004,^12^ Ubink and Quan (2008) find that the traditional authorities’ orientation has been to use the CLSs to consolidate the stool's centralized control over leasehold transactions across much larger areas, and to facilitate the conversion of land held by indigenous farmers to leaseholds for outsiders. In areas with migrant farmers, CLSs were also seen as a way to convert secure tenure arrangements of migrants. These were created through long‐established oral, and sometimes written, sharecropping contracts with landholding families, arguably equivalent to land purchases, for fixed‐term leaseholds subject to rent collection and eventual discretionary renewal by the CLS (see Alden Wily and Hammond, 2001; Amanor and Diderutuah, 2001).

In contrast with LAP's goals of enhancing chiefs’ accountability and the ability of indigenous and tenant farmers to retain their land or to negotiate higher levels of compensation in cases where the traditional authority redevelops or disposes of the land, powerful chiefs saw CLSs as an opportunity to restore and extend their political and economic control over land (see World Bank, 2003). They sought to use CLSs — and the opportunities they provided for centralizing land transactions management and recording and formally documenting land rights — as instruments for land disposals by the elite, by concentrating on facilitating and documenting new land transactions, and failing to document the rights of indigenous and migrant land holders. Ubink and Quan (2008: 210) conclude:

CLSs which strengthen the political and economic weight of the traditional authorities by providing formal recognition of their powers to administer and allocate land … would sanction their ability to generate substantial profits from the disposal of land, over which the original land users exert legitimate claims. If government does not clearly spread the message of the legitimacy of communal interests in land, as recognised by the courts, and the need for accountability of the chiefs, which is stated in the constitution, then it will be providing de facto support for chiefs’ claims that they can convert land in which community members have usufructuary land rights. … This will have the perverse effect that people are disenfranchised rather than empowered.

Studying the CLS's functioning in Wa, Biitir and Nara (2016) come to comparable conclusions. In the north of Ghana it is *tendamba*, first settlers and heads of their families, who are recognized as the customary land authorities. While recognizing certain gains in land administration efficiency and dispute resolution brought about by the CLS's introduction, Biitir and Nara (ibid.) describe how tendamba are not interested in LAP's goal of promoting good governance, but rather see the CLS as a way to centralize control over land and reap financial gains in the form of ground rent. The authors point to a lack of consensus and understanding of the LAP objectives due to inadequate consultation processes. They also highlight that forming the CLS intensified struggles over who are legitimate tendamba and between various sections of the family over the right to allocate land and reap the profits in the form of ground rent. Families fear elite co‐optation by tendamba, through formalizing land authority in the CLS. The article furthermore describes serious shortcomings of the CLS in Wa regarding transparency and accountability.^13^ Anaafo (2015) is similarly critical about the CLS in Nkoranza South municipality. ‘The reformed system’, he writes, ‘puts excessive regulation into the hands of chiefs and this is fading into confiscation of ownership, a practice unintended under customary land tenure relations’ (ibid.: 545). The negative assessment of LAP was shared by the donors, as evidenced by the fact that, in August 2009, DFID withdrew its financial support to the CLSs (see Bugri, 2012).

## CUSTOMARY LEGAL EMPOWERMENT FOR SMALLHOLDERS AND WOMEN

Researchers and donors concur that the LAP has failed to enhance tenure security for the country's smallholders. In contrast, in a country where both opponents and proponents of gender equality believe that women's rights and customary justice are eternal foes (Becker, 2001), Uukwambi Traditional Authority received a remarkable measure of success in enhancing women's position in customary justice. What lessons can we draw from these illustrative cases regarding the engagement with CJSs?

From the broader literature on customary law, we know that when engaging with CJSs, the nature of these systems needs to be taken into account (Harper, 2011b; Sage and Woolcock, 2006; Ubink and van Rooij, 2011). Customary law is unwritten and has no clear‐cut lawmakers. Customary laws are formed in practice and negotiated in leadership structures, consisting of chiefs and their councillors, and in dispute‐settlement fora. They are also defined vis‐à‐vis external structures, such as the state, with the power to enforce, formalize, or recognize local rights. In this context, it is highly relevant which persons or groups within the customary communities are given, or are able to appropriate, the power to define customary law (Oomen, 2005). Customary justice programming may offer a particularly potent momentum for defining and redefining substantive norms and the power of people and institutions over these processes (Ubink and van Rooij, 2011). This article shows that for groups — whether smallholders or women — to be empowered, they need to be included in those spaces where the law is defined and negotiated. Including women in leadership structures and traditional dispute settlement in Uukwambi means they have become part of the very structures where customary law is defined and negotiated. In unwritten legal systems, it is predominantly through being present at traditional dispute settlements that people get to know the rules and norms, and thus know when their rights have been violated, or when a norm seems to be applied differently by the traditional court in similar cases. Through participation, people also learn the rules of the game: what is regarded as proper behaviour during a court hearing; how to get their voices heard by the traditional court; and which things are negotiable — for in CJSs, decisions, particularly regarding the amount of compensation, are often negotiable (Peters and Ubink, 2015). The LAP triggered a formalization of customary land rights and as such provided a critical moment for (re)negotiating the content of customary law. The failure to include smallholders in the processes of defining the CLSs’ functionality and the customary land rights that were to be protected, led to the elite capture of local land rights.

Looking deeper into these cases, we see that there are several forces at play that may have influenced the approach taken in the two case studies. These include the traditional authorities’ willingness to adapt the CJS; the scope of economic opportunities for traditional authorities in the sector of programming; the traditional authorities’ political power; and their relationship with the government. In the Uukwambi case, the traditional leaders’ willingness to make customary justice more inclusive should be seen in light of the TAs’ involvement in the administrative structures of the apartheid government. It is intricately connected to events at the national political level.

With independence finally arriving in 1990, TAs in Namibia were struggling to remain relevant in the new, post‐independence constellation. Many traditional leaders felt the need to redeem popular support, and improve relations with the government. Women's position under customary law had come under fire from both government and society. As Becker (2001: 240) writes about traditional authorities in northern Namibia, the move towards women's inclusion in customary decision‐making structures and processes ‘may well be part of conscious efforts to improve their standing’.^14^ In northern Namibia, traditional leadership, particularly below the level of the chief, does not offer many lucrative opportunities. This lack of financial incentive to become a traditional leader, or to execute this function in a particular way, makes it easier to reach agreement on different rules of the game.

The initiative for the LAP came from government and donors. While the official goals included protection of smallholders’ land rights, the government of Ghana was not willing to endanger its relationship with the chiefs, and as such did not pressure the chiefs to increase transparency and accountability. As a result, traditional leaders saw the CLSs largely as an opportunity to centralize control over land and maximize revenue (Anaafo, 2015; Biitir and Nara, 2016; Ubink and Quan, 2008). Ghanaian chiefs stand to make handsome sums of money from their administrative powers over customary land.^15^ Despite the official aim of enhancing tenure security for smallholders, the government of Ghana was not willing to antagonize the chiefs by thwarting their profit from the administrative position regarding customary land. Government involvement was limited to formalizing and shoring up the powers of traditional leaders, without paying attention to the power dynamics in any of these three domains. Merely subsidizing TAs with material and technical support for CLSs, providing them with a semblance of state‐backed legitimacy in their administrative roles, and hoping for the best, did not provide the basis of a sustainable approach. In fact, it made it harder for smallholders to claim the sanctity of their usufructuary rights in the land; to ask their leaders to account; and to depose leaders that refuse to do so.

Thus, the approach taken to change the customary system of Uukwambi enhanced women's ability to make use of the system to uphold their rights and obtain outcomes that are fair and equitable. In LAP, on the other hand, smallholders’ ability to do the same was diminished through the programming's approach. The political playing field was conducive to successfully including women in Uukwambi's CJS — the chief was willing to make accommodations to gain popularity and to remain relevant in the newly independent country, and the government feared no detriment from weighing in. In the LAP, smallholders were not empowered because the chiefs — who are strong political actors in Ghana — felt secure enough not to compromise their powers in the very lucrative land sector. Additionally, the government was not willing to antagonize the chiefs by pushing for transparency, accountability, and increased recognition of smallholders’ rights (Ubink and Quan, 2008).

Political context may thus explain the approach taken, or the scope of development actors to induce a government or traditional authorities to take an empowerment approach in a particular programme. This does not mean that an empowerment approach is not possible in Ghana in other fields, nor that this approach will be possible in any field in Namibia. An empowerment approach's feasibility depends on the field of engagement, the interests involved, and the case‐specific power constellations.^16^ This article argues that to engage with CJSs, to the benefit of vulnerable groups, requires a focus on power and issues of participation and representation. When such an approach is not possible due to the political context, there is a serious risk that reforms will not reach the intended beneficiaries from vulnerable groups. An enabling political context forms a necessary precondition for successful programming. While this is obviously hard to programme for, it could and should be carefully considered before deciding whether it is sensible to engage with customary justice programming in a particular location.

## CONCLUSION

This article has shown an increasing engagement in development cooperation with CJSs. The approaches and expectations of donors and development agencies challenge their ability to respond adequately to the complexity and context‐specificity of CJSs. They often unintentionally impact negatively on power differentials within the localities, as in the case of the LAP in Ghana. This article posits that customary justice programming needs to engage more with issues of power and empowerment. Ubink and van Rooij (2011) use the term customary legal empowerment to emphasize the participation and representation of marginalized community members in CJSs and their ability to make use of these systems to uphold their rights and obtain outcomes that are fair and equitable. An emphasis on issues of access, participation and empowerment is perhaps even more important when engaging with CJSs than when dealing with the state legal sector. This is due to the unwritten, negotiable character of customary law. Policy makers too often assume a unified community polity governed by an apolitical community leadership. Powers of definition and administration at the local level are overlooked by a belief in a ‘myth of traditionalism’ (Clarke, 2011: 50). The acceptance or portrayal of the powers of representation and negotiation can profoundly affect power relations in customary communities. This article thus pleads for an orientation on customary legal empowerment.

## Footnotes

1 See Ubink (2011b, 2013) for a more elaborate discussion of this case study.

2 See Ubink (2013) for a discussion of this literature.

3 See Ubink (2013: 110) for some numbers.

4 The Communal Land Boards (CLBs) are institutions established in 2003 in line with the Communal Land Reform Act and tasked, inter alia, with dispute resolution on certain land matters.

5 See Ubink (2011a: 143) for more details. These changes cannot be fully attributed to customary law only. Although land grabbing and renewed payment by widows to the headman to retain the land were first outlawed in the written laws of Uukwambi and other Owambo TAs, provisions with similar effect have been included in the Communal Land Reform Act 2002 (section 26 and 42). See Ubink (ibid.: 143–44) for a fuller discussion of this aspect.

6 See, for a more elaborate discussion of this case study, Ubink (2008a: Ch. 2) and Ubink and Quan (2008).

7 Ghanaian Constitution, Articles 267(1) and 36(8).

8 The customary community is called ‘stool’ in reference to the carved wooden stool that is believed to contain the souls of the ancestors. It is a traditional symbol of chieftainship.

9 Also interview, DFID rural livelihoods advisor, Accra, 27 January 2004.

10 Interview, World Bank natural resource management specialist, Accra, 19 January 2004.

11 Interview, DFID rural livelihoods advisor, Accra, 14 September 2005.

12 In 2009, when the pilot phase ended, 38 CLSs had been established in all 10 regions of Ghana (Arko‐Adjei, 2011).

13 See also Nara et al. (2014).

14 See Ubink (2013) for more details. The struggle to remain relevant and gain popularity may, in the case of the chief of Uukwambi TA, also be informed by the fact that he is not the rightful occupant of the Uukwambi leadership position. In 1932, the colonial government deported King Iipumbu, and selected his son to chair the Council of Senior Headmen. In 1986, he was succeeded again by his son, the current leader Herman Iipumbu, who is commonly referred to as Chief Iipumbu. Since the Uukwambi practise matrilineal succession, the latter is not the rightful heir of the royal lineage of Uukwambi.

15 In 2003, a piece of residential land in the surroundings of Kumasi, the second biggest town in Ghana, was worth an equivalent of US$ 1,500. In comparison, in 2009, the going price for a much larger piece of land for a homestead plus adjacent agricultural fields in Uukwambi was widely set at 800 Namibian dollars, which was then approximately US$ 80.

16 One example where customary justice reform may be successful in Ghana pertains to female traditional leadership. Queen mothers, who function alongside male chiefs and are responsible for women's and children's issues, are increasingly recognized and better organized. See: http://www.telegraph.co.uk/women/life/meet-the-queen-mothers-10000-amazing-women-taking-back-power-in/. Interests at stake here are very different from those in the land sector.
